# Tuberculosis control in the 21st century.

**DOI:** 10.3201/eid0702.010222

**Published:** 2001

**Authors:** K A Sepkowitz

**Affiliations:** Memorial Sloan-Kettering Cancer Center, New York, New York 10021, USA. sepkowik@mskcc.org

## Abstract

In response to tuberculosis (TB) outbreaks in the United States in the late 1980s and early 1990s, U.S. hospitals spent tremendous resources to ensure a safer workplace. A remarkable decrease in nosocomial transmission resulted, along with a decrease in TB cases nationally. Federal standards have been promulgated to ensure a safer work environment for all U.S. workers potentially exposed to TB. However, these measures may prove costly and burdensome and thus may compromise the ability to deliver care.


### Tuberculosis Control in the 21st Century

**Kent A. Sepkowitz**
Memorial Sloan-Kettering Cancer Center, New York, New York, USA

In response to tuberculosis (TB) outbreaks in the United States in the late 1980s and early 1990s, U.S. hospitals spent tremendous resources to ensure a safer workplace. A remarkable decrease in nosocomial transmission resulted, along with a decrease in TB cases nationally. Federal standards have been promulgated to ensure a safer work environment for all U.S. workers potentially exposed to TB. However, these measures may prove costly and burdensome and thus may compromise the ability to deliver care.

A consensus that caring for patients with tuberculosis (TB) posed a risk to health-care workers did not emerge until the 1950s and 1960s, when studies established that *Mycobacterium tuberculosis* infection was transmitted by the airborne route (1). However, occupational transmission received little attention until numerous outbreaks of TB and multidrug-resistant tuberculosis (MDRTB) occurred in U.S. and European hospitals in the 1980s and 1990s (2).

More than 20 health-care workers became ill with MDRTB, and at least 10 died (3). Hundreds of health-care workers may be latently infected with MDRTB and thus represent a large repository at risk for future reactivation of disease. Thus, although the MDRTB and drug-sensitive TB outbreaks in the United States and Europe have largely been controlled, the consequences of these outbreaks are still being felt. This article reviews current approaches to TB control in hospitals and prospects for improved control.

### General Considerations

Efficient control of nosocomial TB is compromised by the same difficulties complicating community control, including an insensitive, slow method of diagnosing active disease; an insensitive, nonspecific method of diagnosing latent disease; and relatively slow-acting, complicated courses of medical therapy. However, enormous strides in hospital TB control were made during the late 1980s and 1990s by using common sense, trial and error, and published guidelines (4-6). Most U.S. hospitals now have TB control programs adequate to deal with current TB levels. Should another epidemic occur, however, these approaches may prove insufficient, as in the mid-1980s when the AIDS epidemic introduced a new group at high risk for active TB.

### Community versus Hospital

At the height of the TB resurgence in the early 1990s, many urban U.S. hospitals reported purified protein derivative (PPD) conversion rates in health-care workers of 3% to 5% (3). A survey of U.S. hospitals conducted by the Centers for Disease Control and Prevention (CDC) found a mean conversion rate of 1.6% (7,8). Most recent studies have demonstrated rates <1% annually. Although some of the elevated conversion rate of the early 1990s resulted from the booster phenomenon, much was due to occupationally acquired infection.

Because the conversion rate is now <1% for most U.S. hospitals, infection control teams can investigate each instance of potential exposure from an infectious source case. Despite thousands of potential exposures, many infection control teams are unable to document tuberculin conversions in exposed staff, suggesting that many PPD conversions are the result of community, rather than occupational, transmission. Supporting this perspective are studies associating zip code or area of residence with PPD conversion, rather than specific hospital occupation or specific exposure (9,10).

In some hospitals occupation is significantly associated with risk for PPD conversion. In studies from New York City (11) and Brazil (A. Kritski, pers. comm.), housekeepers were at particularly high risk, independent of area of residence. The hospitals reporting this finding treated high numbers of patients with TB (>100 per year), increasing risk for nosocomial transmission. In hospitals caring for relatively few cases of TB, however, occupational exposure may indeed be less important than exposure in the home or community.

### The Purified Protein Derivative Test

An active surveillance program must rely on the time-honored tuberculin PPD test, which is difficult to place, read, and interpret. In addition, the sensitivity and specificity of the 19th century test are far lower than those of other modern diagnostic tests. Among criticisms of the proposed Occupational Safety and Health Administration (OSHA) standard (10), perhaps the most compelling is the reliance of a $250 million program on the PPD test.

### The Booster Phenomenon

The booster phenomenon confounds the interpretation of the PPD test, complicating TB control programs (12). The extent of boosting in healthy populations was demonstrated in several CDC-led studies of serial skin testing in otherwise healthy young health-care workers. A surprising number of conversions were encountered at the third and fourth test, even in those not exposed to TB, which suggests that boosting with the third and fourth serial test may be more common than assumed. The dramatic rise in PPD conversion rates in hospitals with outbreaks may result as much from nonspecific boosting as from true nosocomial transmission and acquisition of *M. tuberculosis.* By the same logic, subsequent decreases in PPD conversion rates may result from the

Address for correspondence: Kent A. Sepkowitz, Memorial Sloan-Kettering Cancer Center, 1275 York Avenue, New York, NY 10021, USA; fax: 212-639-4386; e-mail: sepkowik@mskcc.org exhaustion of the booster phenomenon in a population, rather than true reduction of nosocomial transmission.

The booster phenomenon is now minimized in hospitals because the efforts of TB control leaders have resulted in frequent skin testing. As TB case rates continue to decrease, along with concern about nosocomial transmission, more unboosted health-care workers will enter the workforce, setting the stage for pseudo-outbreaks similar to those in the 1980s and 1990s (13). In worker populations with high rates of BCG vaccination, boosting is more common (3,13,14). The strongest argument for maintaining the current 6- to 12-month skin testing programs is the need to continue to minimize the booster phenomenon, rather than the need for heightened surveillance to detect TB transmission.

### Approach to Control

The 1994 CDC guidelines for TB control in hospitals and other health-care facilities (4) have become the basis for all U.S. hospital TB control programs, as well as the proposed OSHA standard (10). TB was controlled in hospitals by implementing numerous control measures within a few months, in addition to improving staff awareness and concern (5,15). Thus, it is impossible to know which intervention is the best or most cost-effective for a hospital with limited resources and a low TB case-rate. That said, the old adage that the undiagnosed case is the one most likely to transmit infection remains useful in establishing priorities for TB control.

The 1994 guidelines divide the implementation strategy into a hierarchy of three approaches. Administrative interventions include those to increase the isolation of persons with suspected cases, development of a hospital-wide TB control plan, and maintenance of an active tuberculin skin-test program for health-care workers. Engineering controls, which focus on how best to handle air, include negative pressure capability in respiratory isolation rooms, placement of UV light fixtures, and installation of HEPA filters.

Personal protective equipment (PPE, masks and respirators) decisions were complicated by the lack of clinically meaningful information to guide decisions. After several years of debate, a relatively cheap and comfortable product, the N-95 particulate respirator, was settled upon and is recommended in the proposed OSHA standard.

### Research Needs

In addition to unanswered questions regarding these three interventions, the problems of PPD's insensitivity and nonspecificity and the long treatment courses necessary for cure further complicate hospital TB control. Cost-effective control of TB may depend on improvement in each of these areas.

### Whom to Isolate?

Prompt diagnosis of probable TB requires at least one of three elements: a compatible clinical presentation; sputum smear revealing acid-fast bacilli (AFB); or a chest X ray suggesting TB. Each of these three approaches, however, is relatively insensitive and nonspecific.

One reason that TB control failed so dramatically during the early AIDS epidemic was the relative nonspecificity of TB symptoms in this population. Weight loss, low-grade fevers, and inanition were often the only complaints, even in patients with active pulmonary disease. In patients with advanced AIDS, the same symptoms may be seen in cytomegalovirus disease, lymphoma, or disseminated *M. avium-intracellulare*. This experience illustrated the variable clinical appearance of TB, particularly in populations with abnormal immune function.

Infection control decisions regarding maintenance of respiratory isolation have traditionally been based on the AFB sputum smear, which has approximately 50% sensitivity for TB diagnosis. Therefore, half of patients with active pulmonary TB (i.e., smear-negative disease) are removed from isolation. The relative contagiousness of patients with smear-negative pulmonary results is unknown, but indirect evidence suggests they may transmit infection. A classic study by Grzybowski et al. defined the tuberculin status of an entire community, stratified according to exposure to persons with TB (16). Of small children living in a household with an adult with AFB smear-negative disease, 6% were tuberculin reactive, compared with 0.7% of unexposed age-matched controls. In recent report, a longitudinal molecular typing study (17) indicated up to 17% of cases of TB in San Francisco derived from a smear-negative source case. Despite these studies, the three-smear rule-out has served hospitals well with only rare problems. A practical approach might be for clinicians to continue isolation only for patients who have initial AFB-negative sputum smears but compelling clinical symptoms and chest X rays.

The use of genetic-based tests to diagnose TB may improve diagnostic sensitivity (18). However, few such tests are useful in smear-negative cases and so are of little use in routine infection control practice. They are appropriate, however, to further classify persons with AFB smear-positive disease.

The chest radiograph is notoriously insensitive as a TB screening tool. Up to 10% of persons with pulmonary TB may have an initially normal chest X ray (19). Although computed tomography is sensitive in identifying many abnormalities, routine chest tomography in patients with potential pulmonary disease is not practical.

### When to Discontinue Isolation?

Discontinuing isolation of patients with known TB often is less important for physicians but of paramount importance to the hospital infection control staff, who need to know when a patient no longer can transmit the tubercle bacillus. Previous work, including studies comparing home versus hospital therapy (20) and comparing outcome according to smear or culture status at discharge (21), is >25 years old and may no longer be pertinent to TB care in the 21st century.

Among time-honored approaches (22), the most common is the practice of considering discharge after 2 weeks of apparently effective therapy. Others wait until the sputum AFB smear converts from positive to negative, which may take 4 to 6 weeks. In areas where drug-resistant TB is common, a more cautious approach might be waiting for at least 2 weeks of smear-negativity or, if MDRTB is documented, for culture negativity.

As important as clinical and smear status are the conditions to which the patient will return. Because TB disproportionately affects poor, homeless, and HIV-infected persons, many TB patients should not return to their previous living conditions until shown to be culture-negative. From an infection control perspective, the question "Where is the patient being discharged to?" is often more pertinent than the question "When can the patient be discharged?"

### Engineering Needs

Providing rooms with negative-pressure ventilation was a formidable task for hospitals in the 1990s, and maintaining these rooms is difficult. Warped door frames, shifts in outdoor wind direction, and leaky window seals may interfere with negative-pressure ventilation. Furthermore, no practical consensus has been reached regarding the number of air exchanges per hour needed to protect workers and other patients.

Many experts advocate other engineering controls such as UV light. Innovative studies are ongoing to define optimal aerodynamics and ventilation and establish (or exclude) the role of UV light in TB control. Certainly its inexpensiveness, practicality, and exportability make it the most attractive alternative, should it prove effective.

### Personal Protective Equipment

A long public debate regarding optimal masks and respirators was waged in the early 1990s, as cost and comfort had to be weighed against patient and worker safety (23,24). Eventually, a practical solution, the N-95 particulate respirator, was agreed upon and is now used in U.S. hospitals. Many infection control programs lost a degree of credibility and good will in hospitals where clinicians resisted accepting uncomfortable masks. Although compliance was achieved, the consequences of forcing staff to follow an unpopular, unproven regulation should not be minimized. The success of other important infection control functions, such as annual influenza vaccination drives and handwashing initiatives, depends as much on good will as on scientific merit. The effort expended to enforce a single intervention may have affected the success of other programs to control nosocomial infections.

An additional problem relating to PPE is the requirement for annual fit-testing of masks. Many health-care workers have learned to expedite fit-testing by pretending not to taste the saccharine used in fit-test checks. In addition, few hospitals can deal effectively with the small subset of employees who cannot be fit-tested successfully. Most continue in their current jobs, using putatively inadequate masks. Given the diminishing resources available to hospitals, annual fit-testing could be replaced by an annual self-assessment health questionnaire to identify workers who need fit-testing.

### The OSHA TB Standard

OSHA determined that the occupational risk for TB warranted a standard to ensure worker protection and, in 1997, issued a working draft (10)—the second time that OSHA has developed regulations to protect against an infectious disease. The first such example was the Bloodborne Pathogens Standard, which has significantly reduced occupationally transmitted hepatitis B nationally. The date for implementation of the TB standard is uncertain.

Many health-care workers in urban hospitals had colleagues who became ill with acute TB infection during the MDRTB outbreaks of the late 1980s and early 1990s. Some have watched colleagues die of this nosocomial disease. Thus, most workers welcome attempts to minimize nosocomial spread of *M. tuberculosis.* Concern has arisen, however, that the OSHA approach, estimated to cost $250 million annually, is not scientifically sound and will not reduce risk beyond the current regulations. The debate about scientific soundness derives from the reliance on the PPD test, which is neither sensitive nor specific, unlike the hepatitis B antibody and surface antigen test on which the bloodborne pathogen standard is based. Furthermore, the death rates used in the cost assumptions appear far in excess of what most centers have seen in the past decade. Finally, the regulations may impose a financial burden on facilities such as homeless shelters and drug treatment centers.

The ultimate goal of the standard, no occupational risk, may not be achievable, even with unlimited resources and a perfect test for latent disease. However, the intention of the OSHA standard (minimizing occupational risk for contracting TB) is worthy and will serve to draw public and employer attention to the larger issue of occupational risk for infectious disease. As additional data emerge, a more practical standard that both protects workers and conserves valuable resources may be developed.

### Conclusions

A great deal about hospital TB control was relearned in the 1990s, as hospitals nationwide struggled to contain outbreaks. We are now faced with the realization that we do not know which of the many interventions were effective. Furthermore, 21st century TB control efforts continue to rely on the 19th-century PPD test and the insensitive sputum AFB smear. It is hard to be optimistic about great gains in TB control in the years ahead, beyond the current cautious, but effective "isolate frequently" approach, as long as programs continue to rely on these inadequate diagnostic tests. For at least the next decade, the decidedly low-tech measures of isolating persons with potential disease, wearing masks, and keeping doors closed in rooms that house potential TB patients will remain the cornerstones of TB control in U.S. hospitals.

Dr. Sepkowitz is head of the clinical infectious disease section at Memorial Sloan-Kettering Cancer Center, where he leads infection control. He has written extensively on occupational infections, particularly TB.

### References

1. Sepkowitz KA. Tuberculosis and the health care worker: a historical perspective. Ann Intern Med 1994;120:71-9.
2. Menzies D, Fanning A, Yuan L, FitzGerald M. Tuberculosis among health care workers. N Engl J Med 1995;332:92-8.
3. Sepkowitz KA. AIDS, tuberculosis, and the health care worker. Clin Infect Dis 1995;20:232-42.
4. Guidelines for preventing the transmission of *Mycobacterium tuberculosis* in health-care facilities. MMWR Morb Mortal Wkly Rep 1994;43(RR-13):1-132.
5. McGowan JE Jr. Nosocomial tuberculosis: new progress in control and prevention. Clin Infect Dis 1995;21:489-505.
6. Blumberg HM. Tuberculosis and infection control: what now? Infect Control Hosp Epidemiol 1997;18:538-41.
7. Fridkin SK, Manangan L, Bolyard E, Jarvis WR. SHEA-CDC TB survey, Part I: status of TB infection control programs at member hospitals, 1989-1992. Society for Healthcare Epidemiology of America. Infect Control Hosp Epidemiol 1995;16:129-34.
8. Fridkin SK, Manangan L, Bolyard Em, Jarvis WR. SHEA-CDC TB survey, Part II: efficacy of TB infection control programs at member hospitals, 1992. Society for Healthcare Epidemiology of America. Infect Control Hosp Epidemiol 1995;16:135-40.
9. Snider DE, Cauthen GM. Tuberculin skin testing of hospital employees: infection, "boosting," and two-step testing. Am J Infect Control 1984;12:305-11.

10. Department of Labor, Occupational Safety and Health Administration. Occupational exposure to tuberculosis: proposed rule. Federal Register 1997;62:54159-308.
11. Louther J, Rivera P, Feldman J, Villa R, DeHovitz J, Sepkowitz KA. Risk of tuberculin conversion according to occupation among health care workers at a New York City hospital. Am J Respir Crit Care Med 1997;156:201-5.
12. Menzies D. Interpretation of repeated tuberculin tests: boosting, conversion, and reversion. Am J Respir Crit Care Med 1999;159:15-21.
13. Horowitz HW, Luciano BB, Kadel JR, Wormser GP. Tuberculin skin test conversion in hospital employees vaccinated with bacille Calmette-Guerin: recent *Mycobacterium tuberculosis* infection or booster effect? Am J Infect Control 1995;23:181-7.
14. Cauthen GM, Snider DE Jr, Onorato IM. Boosting of tuberculin sensitivity among Southeast Asian refugees. Am J Respir Crit Care Med 1994;149:1597-600.
15. Blumberg HM, Watkins DL, Berschling JD, Antle A, Moore P, White N, et al. Preventing the nosocomial transmission of tuberculosis. Ann Intern Med 1995;122:658-63.
16. Grzybowski S, Barnett GD, Styblo K. Contacts of cases of active pulmonary tuberculosis. Bull Int Union Tuberc 1975;50:90-106.
17. Behr MA, Warren SA, Salamon H, Hopewell PC, Ponce de Leon A, Daley CL, et al. Transmission of *Mycobacterium tuberculosis* from patients smear-negative for acid-fast bacilli. Lancet 1999;353:444-9.
18. Catanzaro A, Perry S, Clarridge JE, Dunbar S, Goodnight-White S, LoBue PA, et al. The role of clinical suspicion in evaluating a new diagnostic test for active tuberculosis: results of a multicenter prospective trial. JAMA 2000;283:639-45.
19. FitzGerald JM, Grybowski S, Allen EA. The impact of human immunodeficiency virus infection on tuberculosis and its control. Chest 1991;100:191-200.
20. Kamat SR, Dawson JJ, Devadatta S, Fox W, Janardhanam B, Radhakrishna S, et al. A controlled study of the influence of segregation of tuberculous patients for one year on the attack rate of tuberculosis in a 5-year period in close family contacts in South India. Bull World Health Organ 1966;34:517-32.
21. Gunnels JJ, Bates JH, Swindoll H. Infectivity of sputum-positive tuberculous patients on chemotherapy. Am Rev Respir Dis 1973;108:799-804.
22. Menzies D. Effect of treatment on contagiousness of patients with active pulmonary tuberculosis. Infect Control Hosp Epidemiol 1997;18:582-6.
23. Adal KA, Anglim AM, Palumbo CL, Titus MG, Coyner BJ, Farr BM. The use of high efficiency particulate air-filter respirators to protect hospital workers from tuberculosis. N Engl J Med 1994;331:169-73.
24. Nettleman MD, Fredrickson M, Good NL, Hunter SA. Tuberculosis control strategies: the cost of particulate respirators. Ann Intern Med 1994;121:37-40.
